# Exploring the experience of communication in healthcare settings with parents of children with a rare genetic condition: “It's the more negative ones that you remember”

**DOI:** 10.1002/jgc4.70102

**Published:** 2025-08-20

**Authors:** Lucy Burbury, Samantha Ayres, Jackie Boyle, Heather Renton, Aideen McInerney‐Leo

**Affiliations:** ^1^ Dermatology Research Center, The Frazer Institute The University of Queensland Brisbane Queensland Australia; ^2^ Department of Medicine, Dentistry and Health Sciences University of Melbourne Melbourne Victoria Australia; ^3^ Victorian Clinical Genetics Service Murdoch Children's Research Institute Melbourne Victoria Australia; ^4^ Department of Paediatrics The University of Melbourne Melbourne Victoria Australia; ^5^ Genetics of Learning Disability Service Waratah New South Wales Australia; ^6^ Syndromes without A Name (SWAN) Melbourne Victoria Australia

**Keywords:** communication, diagnosed and undiagnosed rare genetic conditions, genetic healthcare professionals, non‐genetic healthcare professionals, parents

## Abstract

Rare genetic conditions (molecularly diagnosed and undiagnosed) of childhood are typically complex in presentation and natural history. Consequently, the diagnostic odyssey can result in families having multiple interactions with diverse healthcare professionals. The quality of these interactions has been relatively underexplored. Semi‐structured interviews explored various parental experiences of communications with genetics and non‐genetics healthcare professionals in Australia. Fourteen parents (12 mothers and 2 fathers) of children with rare conditions (12 diagnosed and 2 undiagnosed) agreed to participate. Each interview was recorded, transcribed, and explored using reflexive thematic analysis. Six themes pertaining to the parental experience of communication around genomics were generated. The first is “the need for family‐centered care” where parents described the importance of involving their child in discussions, even when they are non‐verbal. Second, “the value of à la carte communication”, highlighted that respectful communication, tailored the needs of each family, was highly valued and improved understanding. The third and fourth themes were “technical language is overwhelming” and “negative word choices can be ‘soul destroying’,” respectively. These themes captured how insensitive and/or overly complex discussions, can have negative and lasting effects. The fifth theme, “all results (diagnostic and non‐diagnostic) are significant,” underscored the profound emotional impact of receiving genetic results. The final theme, “where to from here?” revealed that many parents felt abandoned after receiving genetic results and were left to “sink or swim.” Only half of participants had seen a genetic counselor and none could recall being referred to support groups. In conclusion, this study emphasizes the power of language, empathy, and clear, respectful communication for families affected by rare conditions. Additionally, it highlights that these families should have access to genetic counseling and receive referrals for practical and emotional support regardless of whether their child has a molecular diagnosis.

**What is known about this topic**

The diagnostic odyssey is typically protracted in rare genetic conditions, resulting in parental frustration and distress. In healthcare generally, respectful collaborative clinician‐patient relationships and communication promote autonomy, understanding, satisfaction, and trust. Few studies have explored the experiences of communication with genetic and non‐genetic healthcare professionals for parents of children with rare conditions (diagnosed and undiagnosed).

**What this paper adds to the topic**

The receipt of a non‐diagnostic test result, especially from genetic services, was reported as “devastating.” Parents often struggled to understand genetic terminology and appreciated clinicians who communicated concepts clearly, ideally with visual aids. Half of participants had not been referred to genetic counselors, and none could recall being referred to available support groups.

## 1 INTRODUCTION

By definition, a rare condition affects <1 in 2000 people (Zurynski et al., 2008) and collectively affects approximately 8% of the Australian population (Elliott & Zurynski, 2015). Rare conditions typically present in childhood, are often complex, can be life‐limiting, and can involve a range of intellectual, behavioral, and/or physical differences (Baumbusch et al., 2018; Zurynski et al., 2017). The complex and often unique nature of these conditions frequently results in extensive medical investigations and multiple clinical encounters. A third of Australian children with rare conditions see six or more specialists before receiving a diagnosis (Zurynski et al., 2017). This “diagnostic odyssey” is often characterized by protracted periods of parental frustration, uncertainty, and despair (Carmichael et al., 2015; Clark et al., 2024) and sub‐optimal discussions with healthcare professionals (HCPs) (Callander, 2018; Miller, 2021). Between 31 and 58% of children with suspected genetic conditions receive a molecular diagnosis (Mergnac et al., 2022; Stark & Ellard, 2022). Thus, a substantial number of children remain undiagnosed.

Effective HCP‐patient communication is a fundamental aspect of healthcare and is central to providing safe, person‐centered care (Ha & Longnecker, 2010). Over the last 50 years, there has been a shift away from the traditional directive, authoritarian approach (Lee et al., 2020). Person‐centered healthcare requires the clinician to engage collaboratively using clear, respectful communication which promotes patient autonomy, understanding, satisfaction, and trust (Chandra et al., 2018; Geyer, 2021; Levinson et al., 2010; Rogers & Carmichael, 1942). Few studies have evaluated parental experiences of interactions and communications with genetic healthcare professionals (GHCPs) or non‐genetic healthcare professionals (N‐GHCPs) in the context of providing genetic testing and information. It has been posited that consumer comprehension of genomics information is influenced by health literacy levels and emotional responses to information (Lea et al., 2011).

Research focused on parental experiences with clinical genetics services has explored the impact of a prolonged diagnostic odyssey (Zurynski et al., 2017), the largely negative experiences with diagnostic disclosure (Ashtiani et al., 2014), or the experiences of parents navigating the healthcare systems, often as care coordinators (Baumbusch et al., 2018). A recent scoping review on parental needs in the rare disease space identified 11 papers specifically focusing on genetic services, which emphasized the importance of the parent‐clinician empathic connection and ongoing relationship (Crellin et al., 2023). There has been less research exploring the nature and impact of communications around genomic testing, occurring within or outside of clinical genetics departments. This is important given the increasing utilization of genomic testing in mainstream settings. There has also been less research on the impact of these interactions on families where no diagnosis has been made. Thus, this study conducted interviews with parents of children with diagnosed or undiagnosed rare conditions to explore positive and negative communication experiences surrounding genetic and genomic testing.

## 2 METHODS

This study was approved by the Royal Children's Hospital Melbourne Human Research Ethics Committee (HREC) [#91907].

Study aim: To capture the experiences of communication with genetic and non‐genetic healthcare professionals for parents of children with rare conditions (diagnosed and undiagnosed).

### 2.1 Study design and recruitment

Participants were recruited through Syndromes Without A Name (SWAN) Australia, the primary support group for individuals, parents, and families affected by rare genetic conditions (diagnosed and undiagnosed) in Australia. The study was advertised in the closed Facebook group, monthly newsletter, and on their website. Individuals who expressed interest by email were provided with a participation information statement and consent form (PICF). All participants who provided consent by returning a completed copy of the PICF were deemed eligible to participate, and an interview was scheduled. Recruitment ended when LB and AML determined that the final interviews contributed sufficient information power through dense sample specificity and strong dialogue to address the narrow research aim (Malterud et al., 2016).

### 2.2 Procedures

Semi‐structured interviews were conducted via Zoom or telephone by female author LB between March and October 2023. At the time, LB was a genetic counseling student with no children and with limited experience working with families affected by rare genetic conditions. The first interview was conducted alongside experienced genetic counseling researcher AML, who has two adult children and extensive clinical and research experience working with families navigating rare conditions. The interviews lasted from 16 to 58 min, with an average duration of 37 min. Interviews followed a semi‐structured guide that contained prompts for asking about the participants' child/children's diagnostic odyssey, participants' experiences of communication (viewed as both positive and negative) with both genetic and non‐genetic HCPs, and advice to HCPs working with similar families in the future (Appendix S1). The interview guide was developed by LB in collaboration with the supervisory team. Discussions were guided by participants' responses to the open‐ended questions, and additional clarification questions were employed to encourage further elaboration. Interviews were audio recorded, de‐identified, and transcribed by LB. Recordings, identifiable participant information, and de‐identified transcripts were stored on a secure drive at the Murdoch Children's Research Institute. Interview recordings were deleted once the transcriptions were completed.

### 2.3 Data analysis

Our research was conducted within a constructivist paradigm, which assumes that reality is socially constructed and shaped by individual experiences (Wainstein et al., 2023). We used reflexive thematic analysis (RTA) to evaluate the interview transcripts, chosen for its iterative coding, researcher‐driven theme development, and construction of shared meaning through deep data engagement (Braun & Clarke, 2021). The positionality statement and the Reflexive Thematic Analysis Reporting Guidelines responses are available in S2 and S3, respectively. RTA involved an initial phase of familiarization, with LB reading and re‐reading transcripts while making analytical notes on patterns and insights. The entire dataset was then systematically coded using succinct labels to capture meaningful features, incorporating both semantic and latent coding to capture explicit content and underlying conceptual insights (Braun & Clarke, 2019). In collaboration with AML, codes were refined through multiple rounds of analysis, facilitating the construction of broader patterns of meaning. A collaborative analytical approach was used to support LB as an early‐career researcher, with themes developed and reviewed iteratively to ensure coherent and compelling interpretation of the data (Braun & Clarke, 2019). All transcripts were imported into QSR International's NVivo R1 (2020) qualitative data analysis software and were coded using accepted coding techniques (Coffey & Atkinson, 1996). To ensure the confidentiality of participants, patients, and clinicians, illustrative quotes were presented using pseudonyms (chosen by LB) and gender‐neutral pronouns.

## 3 RESULTS

### 3.1 Participants

In total, 14 parents (from different families) were interviewed, including 12 mothers and 2 fathers. Where participants had more than one child with the same condition (4/14), the eldest child was the focus of the interview, as the diagnosis in the first affected child was likely associated with a more protracted diagnostic odyssey and a commensurate number of clinician encounters. Participants first suspected their child may have a medical condition in the first (11/14) or second (2/14) year of life. One of the 11 cases presenting prior to 1 year of age was a prenatal presentation. The age at which genetic diagnosis was made varied significantly from a few days after birth to 20 years of age, with two children undiagnosed at the time of the interview. Clinical presentations of the children varied but broadly included intellectual and/or physical disability, often in the presence of developmental delay, communication difficulty, or epilepsy.

### 3.2 Types of healthcare professionals

**FIGURE 1:**
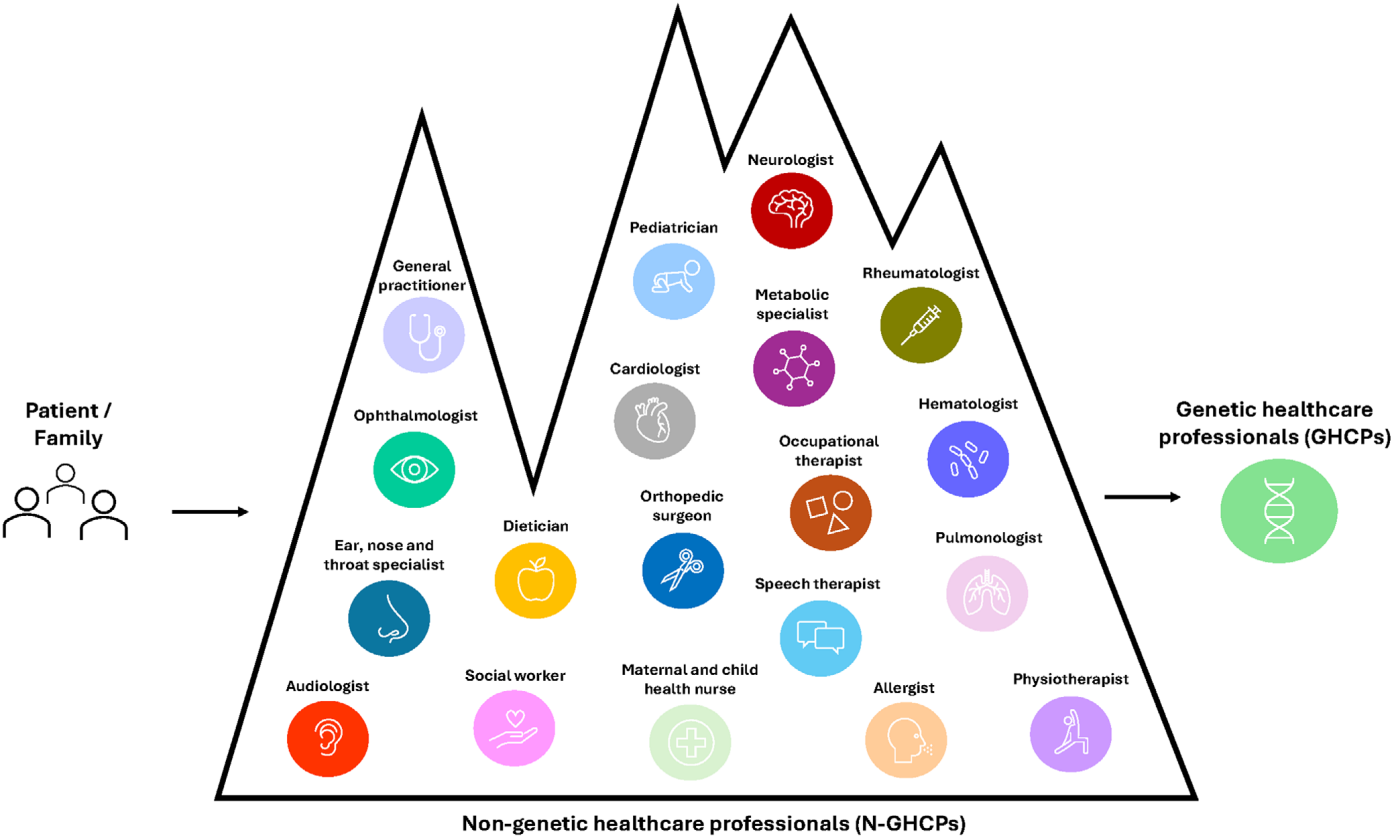
The “mountain” of healthcare professionals encountered by families prior to genetics referral.

The diverse HCPs mentioned during interviews are summarized in Figure 1. They are classified as genetic healthcare professionals (GHCPs), including both genetic counselors and clinical geneticists, or non‐genetic healthcare professionals (N‐GHCPs). On average, parents consulted 13 N‐GHCPs before being referred to a genetics service.

### 3.3 Themes

Six themes were generated regarding parental experiences with communication surrounding genetic testing and diagnoses. These themes captured the importance of personalized and continuous communication, the need for clear and respectful language, and the lasting impact of words used in clinical settings. Parents reflected on the significance of all test results, including non‐diagnostic results, and highlighted the emotional weight of these findings. Finally, families often described feeling isolated in their journey, emphasizing the crucial role of genetic counselors in providing ongoing support.

#### 3.3.1 Theme 1: The need for family‐centered care

Parents emphasized the power of acknowledging and including the affected child within the appointment.

I think it's really important that they acknowledge the child as the child, not as the diagnosis or case…even though they might be non‐verbal…you can still, you know, direct some language towards them. (Fran, Mother)

Most parents interviewed reported feeling like GHCPs specifically were more likely to value their child and treat them like an individual.

In the genetic unit itself, I felt like [they were] special. (Fran, Mother)

Parents appreciated it when HCPs took extra time in appointments, demonstrating a genuine commitment to addressing their needs.

We have had some doctors that…really did always take…the extra time with [Blake] and…went a long way to kind of explore things with us…. (Brad, Father)

It was particularly valued when HCPs provided continuity of care.

[N‐GHCP has] been around for the whole thing…[they] didn't give up…[they're] like a bull at the gate.

#### 3.3.2 Theme 2: The value of à la carte communication

Respectful and inclusive language was highly valued, particularly when discussing sensitive information.

[GHCP] has…been very respectful and listened…[Alex] had dysmorphic features, but [GHCP] explained what those were and…how they are just [Alex] to us and very cute…I felt that [it was] done in quite a respectful manner. (Fran, Mother)

Parents described their appreciation for clinicians who were able to communicate complex concepts in lay language.

…most of it was pretty simple to understand…[GHCP] clarified anything that we didn't understand and…[they] aimed it at exactly right, at the right level of what we could understand, not having much previous knowledge about genetics… [they weren't], um, highfalutin. [They] didn't use a lot of academic medical knowledge. [They were] quite happy to explain it, clarify and explain it again. (Grace, Mother)

Some parents interviewed also found it helpful when GHCPs used visual aids as part of their explanation.

I think one or two doctors sort of drew some diagrams of how, you know, DNA works and stuff like that, which was good and interesting. (Tim, Father)

Additionally, parents emphasized the importance of HCPs tailoring information to meet the needs of each family, particularly by providing clear plans and written summaries that families could reflect on afterward and provide to others involved in their child's care.

Trying to give information that empowers the family…giving solid plans, solid advice…giving summaries of big conversations in writing, getting something that they can take away and reflect on…I think would be really helpful. (Fran, Mother)

#### 3.3.3 Theme 3: Technical language is overwhelming

Parents felt that the use of complex terminology often hindered their ability to understand the information and was a source of additional stress.

…the doctors, they often did often use technical words…that reminded me of a science lesson from high school, quite often…you felt a bit, uh, worn out after going there just because it was quite intense information. (Tim, Father)

Parents reported leaving consultations without a proper understanding of the information discussed.

I just got this genetics, uh, like, you know, print up…basically, you don't understand any of it and um, away you go. (Sara, Mother)

Some parents received a letter that had additional information that they did not recall being discussed during their appointment.

What I heard in that meeting and what has then been written in the medical letter, because it's very medicalized, is quite different. Um, so I think, you know, I had to then make a phone call to the nurse to go, okay, this is what I've interpreted, this is what I heard, is this right? (Fran, Mother)

#### 3.3.4 Theme 4: Negative word choices can be “soul destroying”

Parents repeatedly shared that the use of insensitive and dismissive language had a lasting impact.

It changed me…I became this little ball that used to crawl up in the night…it's soul destroying…how professionals can have that impact, a lifetime impact on you on those first words. (Ava, Mother)

This made it challenging for some parents to recall positive experiences.

…there's obviously probably been a great deal of, you know, really positive interactions that…just kind of blend in. It's the more negative ones that you remember, yeah. (Brad, Father)

The language used by HCPs often had a profound and lasting impact on parents, with many recounting experiences of minimal support and empathy.

[N‐GHCP] said…they'd found that [Charlie] had this [genetic diagnosis]… I was really shocked and sort of started spiraling from that. And I'm like what's the outcome?…What do I do? And [they] just mouthed to me ‘not good’ and then [they] told me to go home and Google it…and that was it…no follow up, no nothing…I left [them] thinking, oh my God, [Charlie is] gonna die in the next 2 years. Like, it was not good. It was atrocious. (Inez, Mother).

This mother (Inez) and others emphasized the importance of having results explained in accessible language so they could be easily understood; expressing how detrimental it can be when this is lacking.

I'll admit things got pretty dark in those 10 days (post‐diagnosis). I would think about how I was going to commit suicide if [Charlie] died…But the stupid thing is…all it took was for someone to explain it to me, which is thankfully what happened when I saw [N‐GHCP] 10 days after. (Inez, Mother)

Parents also remembered comments that devalued or stigmatized their child.

…at 5 months I had an appointment with [N‐GHCP] who told me I should stop going to mother's groups…because my child is too different. (Nina, Mother)

One parent described how this devaluation of their child's life led to the clinician making assumptions regarding family planning.

Our genetic counselor…because at this point, I was pregnant again when the results did come back with the condition that the children had. [They] apologized to us that they hadn't been able to find the results sooner…so that we could have terminated the pregnancy, which was completely not what we would've done anyway…I remember it to this day, it was a very upsetting and still boggles the mind. (Anne, Mother)

#### 3.3.5 Theme 5: All results (diagnostic and non‐diagnostic) are significant

Parents consistently shared that the way their child's genetic test results or diagnosis was communicated felt inadequate or dismissive.

It was a bit of an um, blasé experience…obviously it's a bit of a shock cause there's no forewarning that there would be any groundbreaking news or diagnosis issued, [given] they knew how important a diagnosis was to us. (Tim, Father)

Some parents even received results by mail; with no formal discussion to explain or contextualize the outcome.

We finally got the results and… I got sent a letter [that said] ‘oh no, nothing showed up’…it was so dismissive…they knew that I was really hoping for some results and to just be sent a letter out of nowhere, it was devastating. (Edith, Mother)

Many parents felt that HCPs failed to acknowledge the emotional weight of not having a diagnosis or definitive result.

…no result for families like ours is…almost as significant as a result…it's a devastating outcome, you know? (Elle, Mother)

#### 3.3.6 Theme 6: Where to from here?

All parents interviewed commented on the perceived value of adequate support and follow up, particularly following receipt of molecular genetic test results.

[GHCP] said, you'll probably go home and then you'll have more questions and feel free to contact me about that…it was a lot of work for [GHCP], I'm sure…but it helped us…it built a trust with [them]…[they] never made us feel like just a number. (Cleo, Mother)

Parents consistently reported a lack of support or follow‐up from HCPs following the delivery of their child's diagnosis or test results.

We did receive our results…and there was no real support or follow up from that…we were on our own, so we had to sink or swim. (Anne, Mother)

None of the families interviewed could recall being provided with information about available peer support groups.

…there wasn't really a referral into a support group or anything like that…not solid things, not things that I could take away and digest all the information… (Fran, Mother)

This sense of abandonment often contributed to the distress experienced by many families.

…that abrupt ending probably leads, well adds to the stress and trauma of it all… you're dealing with someone's life and it's not like the issue is solved, it's not like you've actually diagnosed and then fixed the condition if makes sense. The diagnosis actually opens more of a can of worms essentially in this case. (Tim, Father)

Some parents highlighted the value in being able to speak with a genetic counselor.

We were put in touch with a genetic counselor at the time…I have to say [they were] amazing…[They] went above and beyond. (Clare, Mother)

Although half of the families shared that they were not afforded this opportunity.

…interestingly along the way we've never been offered genetic counseling. (Tim, Father)

For families who did have genetic counseling, most parents felt that it helped them understand both the genetic information and the emotional impact.

[GHCP] was more than happy to stay…and help me understand what the emotional aspect of this was and actually getting some more information…[they were] quite supportive and that was quite helpful…because none of it had been easy to understand and none of it was easy to deal with. (Nina, Mother)

In this complex and often uncertain landscape of genetic information, parents expressed the need for guidance on what steps to take next; even in the absence of definitive answers.

I just think with genetics, I guess they don't know the answer, but at least give some avenues. (Sara, Mother)

## 4 DISCUSSION

This study aimed to explore the experience of communication in healthcare settings with parents of children with rare genetic conditions (diagnosed and undiagnosed). Parents were invited to share both positive and negative experiences of communication with GHCPs and N‐GHCPs to allow for a broad and thorough understanding of current communication practices in Australia. This study found that inclusive, clear, and respectful communication that incorporated simple, tailored explanations helped alleviate stress and enhance understanding. Equally, participants described that insensitive, complex, and dismissive communications can have a lasting impact and may cause individuals to feel disrespected and overwhelmed. Themes that have been rarely reported in the genetics literature to date include parents describing feelings of abandonment following receipt of genetic results, and that all results, whether diagnostic or non‐diagnostic, are significant. These findings have important implications for all HCPs working with families affected by rare conditions.

Parents desire sensitive, genuine, and compassionate interactions with HCPs, and consequently value HCPs who display humility, empathy, and a commitment to the wellbeing of their child. These findings reflect similar studies on the parental experience which suggest three ideal characteristics of effective communication, namely displaying respect and consideration, offering emotional and informational support, and encouraging individual reflection in the context of their situation (Gómez‐Zúñiga et al., 2019; Heller & Solomon, 2005; Hsiao et al., 2007). Treating parents with sensitivity and thoughtfulness, and ensuring they feel “cared for” at every stage of their child's diagnostic odyssey and beyond, is crucial for fostering trust in the parent‐clinician relationship (Crellin et al., 2023; Gómez‐Zúñiga et al., 2019). This desire for family‐centered care is not novel, as it acknowledges the needs of both children and their families in the care process. One study explored the impact of family‐centered care intervention and found that parents felt empowered to prioritize their role as caregivers and advocates, while children valued opportunities for inclusion and meaningful engagement (Fratantoni et al., 2022). Similarly, parents in this study emphasized the importance of acknowledging and involving their child as an individual, even when they are non‐verbal. This frustration is also felt by individuals with intellectual disabilities who call for more respectful and inclusive consultations with HCPs (Dunn et al., 2024; Strnadová et al., 2023). Additionally, parents spoke of the importance of using appropriate and accessible language to describe their child's condition and abilities, rather than focusing solely on their disabilities (Ashtiani et al., 2014).

Parents value HCPs who use accessible language and terminology when explaining their child's condition, prognosis, and management. Clear, effective communication has been shown to improve patients' understanding and overall satisfaction with HCPs (Ashtiani et al., 2014; Demarest et al., 2022; Hsiao et al., 2007). Parents felt that visual aids (i.e., diagrams or videos) enhanced the communication process. Consistently, prior research has shown that visual aids facilitate comprehension (Hammond et al., 2021; Reiff et al., 2012). The study participants emphasized that making communication accessible and individualized improved effectiveness, which reflects research showing that optimal communication requires clinician awareness of parents' knowledge and capabilities to maximize comprehension (Dellve et al., 2006; Hallberg et al., 2010). Furthermore, parents expressed difficulty in understanding complex medical terms and genetic information. The literature shows that technical and medicalized terminology, especially when HCPs dominate the session, can hinder parents' understanding of information and contribute to parental distress and uncertainty (Ashtiani et al., 2014; Chapple et al., 1997; Reiff et al., 2012; Waxler et al., 2013). This may reflect clinicians' lack of confidence in navigating complex or emotional conversations (Stephens et al., 2021), potentially due to the inconsistent teaching and evaluation of genomic communication skills training, as highlighted in a recent scoping review (Medendorp et al., 2021). Consequently, resources are emerging to support clinicians in developing these skills (Genomics England, 2022).

The long‐term psychological impact of poor communication generally, and negative communication specifically, was highlighted in this study. Parents also shared experiences in which HCPs left them feeling distressed due to the framing and language used to describe their child's condition. This included offensive language coupled with a perceived lack of empathy, bluntness, and directiveness. Such clinicians were characterized by Gómez‐Zúñiga et al. (2019) as having a “closed profile,” that is, being less skilled or interested in providing companionship, appearing emotionally distant, and less willing to work collaboratively with families or accept suggestions. Poor, insensitive communication may have long‐term psychological impacts on parental coping (Nevin et al., 2022). Parents in this study recounted the profound impact of these encounters. One mother described considering suicide when unclear communication led her to assume that her child was going to die. Other research has emphasized the negative impacts of failing to provide hope or perspective (Ashtiani et al., 2014; Waxler et al., 2013). Unsurprisingly, depression is common in parents of children with severe, chronic conditions, with one Australasian study identifying that 37% of parents reported receiving treatment for depression (Pelentsov et al., 2016). Additionally, parents struggle with grief, feelings of uncertainty or fear, hopelessness, guilt, and a loss of control, identity, and/or purpose (Baumbusch et al., 2018; McConkie‐Rosell et al., 2018; Spillmann et al., 2017). Thus, the psychological vulnerability of parents of children with complex conditions and needs makes sensitive, empathic, and clear communication all the more imperative.

A diagnosis, or lack thereof, was highly significant to the parents of children with rare genetic conditions in this study, and how this was communicated was remembered. Myra Roche, a genetic counselor with experience in rare genetic conditions, commented that parents struggle to forgive and forget when their child's genetic diagnosis was delivered inappropriately (Roche, 2006). Ashtiani et al. (2014) explored the parental experience of receiving their child's genetic diagnosis and reported that 61.5% of parents recalled this experience negatively. This dissatisfaction is commonly attributed to an insensitive communication approach, limited information, and inadequate psychological support (Anderson et al., 2013; Ashtiani et al., 2014; Domaradzki & Walkowiak, 2024). Parents in our study articulated that HCPs often failed to appreciate the enormity of the genetic test result and the potential devastation of diagnostic genetic results, as has been reported previously (Zurynski et al., 2017). Further, participants described the profound, and even “devastating” significance of non‐diagnostic genetic results, which they felt were relayed in a cavalier, dismissive manner as if insignificant or benign. As most research studies focus on the impact of receiving a genetic diagnosis, as opposed to the impact of not receiving a diagnosis, we are not aware of any research which has documented this previously. However, parental anxiety has been shown to decrease following receipt of a genetic diagnosis (Kolemen et al., 2021), which quantitatively indicates the psychological toll of living without a diagnosis.

The lack of support and follow‐up was at the forefront of parental concern with most families feeling like they were left to “sink or swim” following the receipt of genetic test results. This is common in rare genetic conditions where management and referral practices are less obvious or defined (Fitzgerald et al., 2021; Pelentsov et al., 2016; Zurynski et al., 2017). Peer support groups, such as SWAN, offer emotional and practical support by connecting individuals and families with shared experiences, providing information and resources, fostering open discussion and peer support, and empowering members to advocate for better care (Delisle et al., 2017). Despite this, no parents in our study could recall being referred to a support group. This “abrupt ending” reportedly intensified the stress and trauma surrounding their child's diagnosis, particularly in the case of rare conditions where a molecular diagnosis may provide an explanation but not a solution, resulting in ongoing challenges and supportive care needs (Somanadhan et al., 2025). This is consistent with a study showing that receiving a molecular diagnosis for a genetic condition did not end uncertainty, but rather changed it from questions about etiology to concerns about potential severity (Withers et al., 2021). However, parents found some comfort in being able to see genetics experts, even when there was no diagnosis or answer. Lack of patient follow‐up is a long‐recognized problem with clinical genetics services (Esmer et al., 2004), which is unlikely to change in an era when test utilization is increasing, and clinical genetics services are finite. Thus, resource provision and referrals to appropriate clinical and social support services is critical. As one parent put it, “with genetics, I guess they don't know the answer, but at least give some avenues.”

Only half of the parents interviewed had ever been provided with genetic counseling, which is consistent with a study on 462 Australian children living with rare conditions, where only 44.8% received genetic counseling (Zurynski et al., 2017). An early chart review evaluated this further and showed that individuals/families with diagnosed rare conditions were more likely to receive genetic counseling than undiagnosed cases (62% vs. 5%) (Esmer et al., 2004). This may contribute to parents of undiagnosed children feeling particularly abandoned with minimal support to guide them through the immediate and long‐term challenges. Families who received genetic counseling were predominantly positive about that experience, as it provided information regarding their child's condition and emotional support. Genetic counseling has been shown to empower individuals to make informed decisions, improve understanding, and decrease distress through a supportive, empathic, and person‐centered approach (Elliott & Friedman, 2018; Medendorp et al., 2021). This is particularly important for children with rare or undiagnosed genetic conditions, as the limited information compounds the uncertainty, thereby necessitating family‐specific guidance and emotional support (Inglese et al., 2019). Although genetic counselors are expertly trained to provide this type of care (Elliott & Friedman, 2018), the mainstreaming of genomic testing means that fewer patients and families will be referred to genetic counseling in Australia (Cormack et al., 2024). Upskilling N‐GHCP to offer genomic testing can shorten the diagnostic odyssey, but it is important that individuals are referred for additional psychological support and counseling to ensure that parents' questions and concerns have been suitably addressed and to facilitate the coping process.

### 4.1 Practice implications and future directions

This study identified the importance of providing family‐centered care and emotional support alongside the information provision, given the psychological impact of caring for a child with a rare genetic condition. As these families interact with genetics and non‐genetics clinicians, prior to and following a diagnosis, best practice guidelines and education regarding appropriate and effective communication practices should be provided to all HCPs. Additionally, half of the participants in this study had never been offered dedicated genetic counseling, despite the fact that genetic counselors are well placed to provide post‐test support given their specialized training in health and psychological communication. Lack of referrals to genetic counseling has been reported previously, especially in families who have not received a diagnosis. This study suggests that the need for genetic counseling may be as great, or even greater, among families whose children remain undiagnosed. Finally, our findings highlight a lack of follow‐up and support pathways for families following receipt of genetic test results. Thus, guidelines regarding the care of individuals with rare genetic conditions (diagnosed and undiagnosed) should include a recommendation to be referred for genetic counseling, to patient support groups, and to allied health and social support services.

### 4.2 Limitations

Strengths of this study include agnostic ascertainment of individuals who self‐selected to participate. However, the opt‐in nature of recruitment can also be seen as a limitation, as people who had more neutral or positive experiences may have been less motivated to participate (Smith & Noble, 2014). There was a gender disparity in this study as it mostly represented the lived experiences of mothers, with minimal representation from fathers. Participation bias may have affected the nature of the identified themes. Given the variable time lapses between the communications and the interviews, participant recounts are vulnerable to recall bias. This could have affected the number of reported healthcare professionals seen prior to being referred to a genetics service. Additional questions regarding the sequential or simultaneous nature of these appointments would be needed to clarify the patient journey. Finally, the inclusion of HCP perspectives may have provided additional context to better understand the communication challenges described by parents.

## 5 CONCLUSION

The study findings demonstrate the empowering impact of positive, respectful communication and, conversely, the devastating, lasting impacts of insensitive or confusing encounters. The protracted diagnostic odyssey meant that a diagnostic and non‐diagnostic genetic test result held profound significance for these families, and the nature and sensitivity of the communication process should reflect this. Only half of families saw genetic counselors, and none were provided with information about support groups. Participants expressed feelings of isolation, devastation, and abandonment. Guidelines for rare genetic conditions should include recommendations for genetic counseling and the provision of information on available support organizations for all families.

## AUTHOR CONTRIBUTIONS

The project was devised by the Education, Ethics and Social Issues Committee (EESIC) of the Human Genetics Society of Australasia. Lucy Burbury, Samantha Ayres, and Aideen McInerney‐Leo designed the investigation, protocol, and ethics submission. The protocol and interview guide were critically reviewed by Samantha Ayres, Aideen McInerney‐Leo, Jackie Boyle, and Heather Renton. Interviews were conducted by Lucy Burbury. Data analysis and result interpretation were carried out by Lucy Burbury and Aideen McInerney‐Leo. The initial manuscript was written by Lucy Burbury and Aideen McInerney‐Leo, while Samantha Ayres, Jackie Boyle, and Heather Renton provided critical revisions. Lucy Burbury and Samantha Ayres confirm full access to all study data and take responsibility for its integrity and the accuracy of analysis. All authors approved the final version for publication and agree to ensure the integrity of and resolution of any issues concerning the work's accuracy or integrity.

## CONFLICT OF INTEREST STATEMENT

The authors declare they have no conflict of interest.

## ETHICS STATEMENT

Human studies and informed consent: This study was approved by the Royal Children's Hospital Melbourne Human Research Ethics Committee (HREC) [#91907].

Animal studies: The authors of this article did not conduct any studies involving non‐human animals.

## Supporting information


Appendix S1

## Data Availability

De‐identified data supporting the findings of this study can be obtained from the corresponding author upon reasonable request, provided ethical approval is granted by the ethics committee that approved the original study.
